# Driving biomass breakdown through engineered cellulosomes

**DOI:** 10.1080/21655979.2015.1060379

**Published:** 2015-06-11

**Authors:** Sean P Gilmore, John K Henske, Michelle A O'Malley

**Affiliations:** Department of Chemical Engineering; University of California; Santa Barbara, CA USA

**Keywords:** anaerobic fungi, biofuels, cellulase, cellulosome, lignocellulose

## Abstract

Extraction of sugar is the rate-limiting step in converting unpretreated biomass into value-added products through microbial fermentation. Both anaerobic fungi and anaerobic bacteria have evolved to produce large multi-cellulase complexes referred to as cellulosomes, which are powerful machines for biomass deconstruction. Characterization of bacterial cellulosomes has inspired synthetic "designer" cellulosomes, consisting of parts discovered from the native system that have proven useful for cellulose depolymerization. By contrast, the multi-cellulase complexes produced by anaerobic fungi are much more poorly understood, and to date their composition, architecture, and enzyme tethering mechanism remain unknown and heavily debated. Here, we compare current knowledge pertaining to the cellulosomes produced by both bacteria and fungi, including their application to synthetic enzyme-tethered systems for tunneled biocatalysis. We highlight gaps in knowledge and opportunities for discovery, especially pertaining to the potential of fungal cellulosome-inspired systems.

## Abbreviations

CBM: Carbohydrate Binding Module
ELISA: Enzyme-Linked Immunosorbent Assay
GH: Glycoside Hydrolase
GST: Glutathione S-Transferase
SLH: Surface Layer Homology

## Introduction

Plant biomass is an abundant source of cellulose and hemicellulose, which are sugar-rich polymers that can be depolymerized and fermented into value-added chemicals.^1^ Many bioprocessing strategies employ metabolically engineered microbes like *Saccharomyces cerevisiae* or *Escherichia coli* to convert biomass hydrolysates into target products.^2^ However, sugar extraction from biomass relies on energy intensive chemical pretreatment to remove lignin and other recalcitrant biopolymers from substrates prior to hydrolysis.^3,4^ These steps are often performed in concert with expensive enzyme treatments,^5^ which limits the economic feasibility of this approach. Therefore, there is a critical need to develop enzyme systems that can act on unpretreated biomass, especially those that can be produced at high titers by fermentation capable microbes.

**Figure 1.**
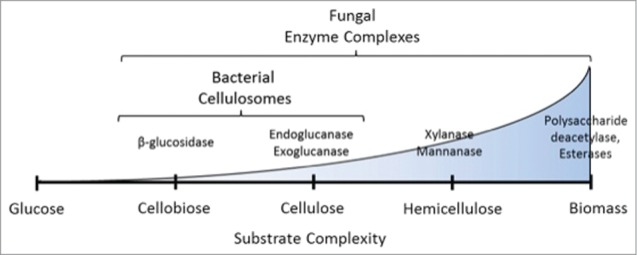
Enzymes required for hydrolysis scale with the complexity of the biomass substrate. A wide variety of enzymes are required to depolymerize the components of crude, unpretreated biomass. For complete conversion of cellulose into glucose, a cocktail of β-glucosidases, endoglucanases, and exoglucanases are required. Hydrolysis of hemicellulose requires enzymes with additional functionality, including xylanases and mannanases. To access these sugar polymers from crude biomass, it is often necessary to solubilize lignin, which is crosslinked within cellulosic and hemicellulosic fibers. For this process, accessory enzymes such as polysaccharide deacetylases, peroxidases, and esterases are required. Bacterial cellulosomes typically contain enzymes required only for cellulose degradation while fungal enzyme complexes contain a richer diversity of enzymes to enable degradation of crude plant material.^7-9^

A wide variety of enzymes with complementary function are required to degrade plant biomass (**Figure 1**). While natural cellulolytic bacteria and aerobic fungi are a rich source of such enzymes, these microbes secrete a limited subset of enzyme types that cannot fully depolymerize crude plant material.^6^ To identify enzymes that degrade crude lignin-rich biomass one must look to the microbes that have evolved to degrade it. For example, large herbivores rely on a microbial consortia composed of anaerobic gut microbes (e.g. bacteria and fungi) to convert grasses and hay into sugar for the animal. Together, these anaerobic microbes secrete powerful enzymes capable of breaking down crude, unpretreated biomass.^7^

The high efficiency biomass breakdown associated with anaerobes stems from their ability to synthesize large multi-cellulase complexes called cellulosomes. These complexes link together all the diverse enzymes necessary for cellulose degradation through a "plug-and-socket" modular interaction via protein domains termed dockerin and cohesin. Logically, these tethered enzyme systems are suspected to increase degradation efficiency by concentrating active sites of the enzymes and targeting them toward the plant material, leading to substrate tunneling of the biomass toward free sugars. The well-studied bacterial cellulosome has demonstrated the power of these modular enzyme complexes for biomass degradation. By comparison much less is known about fungal cellulosomes, yet early research suggests that they have functionalities equal to or greater than bacterial cellulosomes and can also be applied for bioprocessing applications. For example, anaerobic fungi produce a greater diversity of enzymes compared to anaerobic bacteria, including hemicellulases, such as xylanase and mannanase,^8^ other accessory enzymes responsible for lignin reorganization, such as polysaccharide deacetylases and targeted esterases.^9^

## Bacterial Cellulosomes – From Native Parts to Synthetic Designer Cellulosomes

Bacterial cellulosomes were first described in 1983 as "a discrete, cellulose-binding, multi-enzyme complex for the degradation of cellulosic substrates."^10^ They have since been found in many different bacterial species, primarily in the *Chlostridium*,^10^ *Ruminococcus,*^11^ *Acetivibrio,*^12^ and *Bacteroides*^13^ genera. Typically, these complexes in bacteria are built upon a large, non-catalytic protein called a scaffoldin.^14^ The size of bacterial scaffoldin proteins can vary widely, generally from 50kDa to 250kDa^15^; this size variation is related to the number of repeats of cohesin domains included in a particular scaffoldin. The cohesin domains associate strongly with dockerin domains on the individual cellulases,^16^ resulting in full complexes that range in size from 1.5 to 6MDa,^14^ and in bacteria the dockerin-cohesin interaction is highly species specific.^17^ Additionally, the scaffoldin very frequently contains one or more carbohydrate binding modules (CBM) to target the complex to its substrate.^10^ Finally, the entire cellulosome complex associates with the cell surface through anchoring domains called Surface Layer Homology (SLH) domains.^18^ For further information on native bacterial cellulosomes there are several in depth reviews such as those by Bayer et al.^14^ and Doi et al.^15^

Following detailed studies on bacterial cellulose-degrading complexes, the concept of "designer cellulosomes" was first introduced by Bayer in 1994.^19^ Once cellulosomes were recognized to consist of modular parts, Bayer and colleagues proposed utilizing the native scaffoldin or cohesins with heterologous dockerin-fused enzymes to produce artificial cellulosomes, which would amplify cellulolytic capabilities for normally non-cellulolytic systems.^19^ Since then, many different reports have characterized "mini cellulosomes" inspired by bacterial cellulosomes.^20-24^ These studies have demonstrated that enzyme tethered complexes are much better than free enzymes at degrading low-accessibility, highly crystalline, insoluble substrates when produced in recombinant systems.^20-24^ However, very little improvement in activity is observed when complexes act upon well mixed, soluble substrates.^20-24^

**Figure 2.**
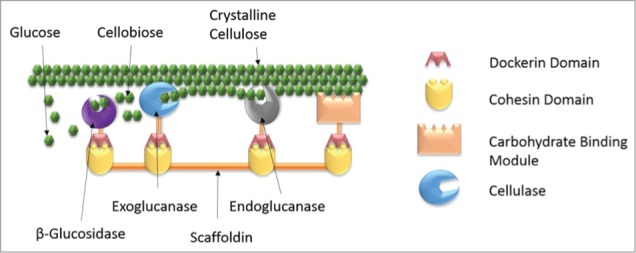
Synergistic Action of Cellulases within a Cellulosome. Cellulases assemble in close proximity on a noncatalytic protein called a scaffoldin. The endoglucanse reduces the degree of crystallinity of the cellulose substrate and liberates 2 cellulose chain ends. The exoglucanase processes along a free chain, freeing cellobiose with each cleavage. This cellobiose is then transferred to a nearby β-Glucosidase, which hydrolyzes it into 2 glucose monomers.

Taken together, these observations suggest that the efficiency of cellulase complexes stems from CBM-facilitated enzyme targeting, as well as the relative organization of the enzymes within the complex. As shown in **Figure 2**, cellulosome complexes are targeted to biomass substrate by the CBM. Once positioned, the cellulases act as a disassembly line to synergistically tunnel reactants and products toward sugars. In particular, endoglucanases reduce the crystallinity of the substrate and free up free chain ends; these ends are then degraded by nearby processive exoglucanases, which release cellobiose as they move along the chain. Tethered β-glucosidases subsequently hydrolyze cellobiose to glucose. Such a model is supported by several reports, which noticed an increased rate of conversion of cellobiose to glucose^24^ and xylobiose to xylose^22^ when a β-glucosidase or β-xylosidase was included in synthetic mini cellulosomes. These results suggest that a mechanism similar to substrate channeling occurs, where the β-glucosidase acts on cellobiose as it is liberated from cellulose by a nearby exoglucanase. Indeed, other reports have demonstrated substrate channeling by fusing enzymes from a metabolic pathway to dockerins, and linking them together on a scaffoldin truncation,^25^ further demonstrating the broad applicability of the cellulosome system to any multi-enzyme biocatalytic process beyond those associated with cellulose degradation.

## Fungal Cellulosomes – Undercharacterized and Heavily Debated Complexes

Although large multi-enzyme complexes have been documented in gut fungi since 1992,^26^ they are woefully understudied compared to their bacterial counterparts. While they are believed to assemble through a modular cohesin-dockerin type interaction,^27^ the identity of the fungal cohesin domain, or a scaffoldin equivalent, remains elusive and is heavily debated. In fungi, dockerin domains are fused to catalytic enzymes, but these dockerins exist in tandem repeats at either the N or C-terminal of cellulases,^28^ compared to single copies often restricted to the C-terminal of cellulases in bacterial cellulosomes.^24^ The specificity of the dockerin-mediated interaction also appears to differ greatly from that found in anaerobic bacteria. Nagy et al.^29^ demonstrated through an ELISA that dockerin from one species can interact with cellulosomes from other species, suggesting that the dockerin-cohesin interaction is not species-specific as it is in anaerobic bacteria. Additionally, several reports estimate fungal cellulosomes to be greater than 1 MDa in size,^30,31^ although they have also been reported to be as small as 334kDa,^31^ and as large as 80MDa.^8^ This is similar to the bacterial system, where the size varies with the number of cohesins and particular type of enzyme associated.

Over 20 years ago, the first reported fungal cohesin was identified,^27^ yet there has not been convincing evidence since to substantiate this finding. At least 4 other reports have challenged this original finding, each proposing other proteins as fungal cohesins.^28,29^^,^^32,33^ By probing denatured fungal cellulosomes with an epitope-tagged recombinant dockerin, several studies have sought to find putative cohesin(s) through a Western Blotting approach.^28,29^^,^ ^32,33^ A short summary of the findings of these papers is detailed in **Table 1**. More recent reports have coupled this effort with Mass Spectrometry to identify the sequence of the interacting cellulosome-associated protein (represented by parentheses in the table).^29,33^ Interestingly, these proteins were all classified as catalytic proteins by sequence homology.^29,33^ In this regard, a catalytic scaffold would hold a distinct advantage over the bacterial scaffolding system because it would eliminate the need for the large, noncatalytic scaffold found in bacterial systems. However, as documented in **Table 1**, the protein identified varied with each study, therefore casting doubt on the results found in all of the studies. Furthermore, the method utilized must be called into question, since the cellulosome protein is denatured during SDS-PAGE before being transferred to the blot. Thus, such a technique is unlikely to fully replicate the native protein-protein interactions within fungal cellulosomes.

**Table 1.** Proteins Speculated as Cohesins within Fungal Cellulosomes

Organism	Putative Cohesin Protein	Method of Detection^*^	Reference (Year)
*Neocallimastix patriciarum J11*	79kDa Protein (GH48)	Dockerin-GST Western Blot	Wang et al.^33^ (2014)
*Piromyces equi*	100kDa Protein (GH3)	Dockerin-GST Western Blot	Nagy et al.^29^ (2007)
*Orpinomyces sp. strain PC-2*	64kDa Protein	CelC-Dockerin Western Blot	Steenbakkers et al.^28^ (2001)
	66kDa Protein		
	95kDa Protein		
	130kDa Protein		
*Piromyces equi*	97kDa Protein	Dockerin-GST Western Blot	Fillingham et al.^32^ (1999)
*Piromyces equi; Neocallimastix patriciarum*	97kDa Protein	Dockerin-GST Western Blot	Fanutti et al.^27^ (1995)
	116kDa Protein		

* Dockerin-GST Western Blot signifies a Western blot performed with a recombinant dockerin expressed as a fusion to Glutathione S-Transferase (GST) as the primary probe, and an Anti-GST antibody as the secondary probe. CelC-Dockerin Western Blot signifies a His-tagged Glycoside Hydrolase 6 cellulase (CelC) with its native dockerin domains was used as the primary probe, with an Anti-His antibody as the secondary probe.

It was suggested by Nagy et al^29^ that the fungal cellulosome interaction might be mediated by dockerin binding to post-translational modifications on the cohesin, which would not necessarily require a folded protein cohesin motif. They supported this claim with evidence that the cellulosomal proteins might be glycosylated, although they could not identify the exact nature of the glycans. However, this claim contradicts the findings of Raghothama,^34^ who identified several residues important for binding through an ELISA with mutant recombinant dockerins against native cellulosomes. These residues were aromatic amino acids (Tryptophan and Tyrosine), with flat edges of the aromatics presented as the likely interacting regions.^34^ Such regions are more indicative of protein-protein interaction than protein-glycan or other post-translational modifications.

Although much is still unknown regarding fungal cellulosome composition and structure, there are some preliminary findings from fungal cellulosomes that suggest that they may have distinct advantages over bacterial cellulosomes. The major degradation product of fungal cellulosomes is glucose, compared to cellobiose from bacterial cellulosomes.^8^ This is an attractive feature, since it removes the need to supplement costly β-glucosidases to cellulosomes. Two distinct classes of β-glucosidases have been identified in anaerobic fungi: freely diffusive (those without a dockerin domain)^35^ and cellulosome associated (with a dockerin domain).^36^ Finally, the enzymes identified to date from fungal cellulosomes comprise a long list with a diverse array of substrate specificities. A recent review by Haitjema et al.^37^ contains a complete list of glycoside hydrolase families and the species from which they were identified. There are close to 30 separate families represented across the various genera, which again reflects the large number of enzymes required to fully hydrolyze lignocellulose as demonstrated in **Figure 1**, indicating that fungal cellulosomes likely harbor complementary functions to their bacterial counterparts.

## Opportunities for New Discoveries and Synthetic Fungal Complexes

While much has been learned about anaerobic fungi since they were first reported by Orpin in 1975,^38^ there is still a great deal of information that remains elusive, particularly regarding the cellulose-degrading complexes produced by the fungi and the sequence information encoding these enzymes. With the advent of powerful techniques, such as Next Generation Sequencing (NGS) and Mass Spectrometry, many of the mysteries regarding the fungal cellulosome should now begin to unfold. The most important information precluding our understanding of fungal cellulosomes is the identity of the cohesin and scaffoldin protein, including the conservation of these domains across fungal genera. Once known, it will undoubtedly become easier to determine the size, architecture, and potential diversity of anaerobic fungal cellulosomes. Finally, this knowledge can be applied to creating synthetic systems using the fungal cohesins and dockerins to tether recombinant enzymes, which likely have desirable attributes distinct from those inspired by anaerobic bacteria as described above.

One exciting hypothesis to explain the wide range of size and compositional heterogeneity in fungal cellulosomes is that smaller cellulosomes associate into larger polycellulosomes, as has been demonstrated in some anaerobic bacteria.^8^ Therefore, beyond just finding the identity of the cohesin domain, it is important to determine the architecture of the cellulosome and the possible mechanism for formation of polycellulosomes. Similarly, it is important to determine whether certain cellulases are positioned specifically within the complex, and what factors drive this specificity – for instance, how the complex evolves as a function of its lifetime. Such information could inform the development of smart "tunable" cellulosomes that adjust their composition and enzyme stoichiometry as a function of their substrate.

While the biological reason for tandem dockerin motifs in fungi is still unknown, it might be the key mechanism controlling spatial positioning of enzymes within native fungal complexes, which can be exploited to build synthetic complexes. There has been evidence to suggest that the binding affinity within fungal cellulosomes relates to the number of dockerin domains present in docked enzymes.^29^ However, it is also possible that the repeats lead to greater specificity within a targeted location in the scaffold, which can be exploited in fungal cellulosome-inspired complexes to guide dockerin-fused enzymes to a targeted position. Given the sequence divergence of fungal dockerin domains compared to those from bacteria, fungal cohesin-dockerin assembly is also likely governed by entirely different interactions, which will undoubtedly be useful for numerous synthetic biology applications that direct tailored protein-protein interactions.

In conclusion, there is still much to learn about the cellulase complexes produced by anaerobic fungi. Compared to their bacterial relatives, fungal cellulosomes are capable of completely converting crude lignocellulosic biomass to its component sugars, due to the wide range of enzymes encoded within the complex. At the very least, they are an attractive resource for discovering new biomass degrading enzymes, novel modular protein-protein interaction domains, and potentially new enzyme superstructures from nature. Beyond this, their characterization could soon reveal a novel scaffolding system, which has applications in creating synthetic fungal enzyme complexes, as well as inspired complexes for any set of tandem biocatalytic processes.
